# A Comparative Study of Dynamic Hip Screws and Proximal Femoral Nails in Intertrochanteric Fractures

**DOI:** 10.7759/cureus.59063

**Published:** 2024-04-26

**Authors:** Varun Thusoo, Ashish Nehru, Sachin Kudyar, Arjun S Chakrapani, Eshaan Singh Saini, KV Alok, D Narender, Suraj Philip

**Affiliations:** 1 Orthopaedic Surgery, Adesh Medical College and Hospital, Ambala, IND; 2 Orthopaedics and Trauma, Government Medical College and Rajindra Hospital, Patiala, IND; 3 Orthopaedics, Government Medical Hospital, Jammu, IND; 4 Orthopaedics, Apollo Speciality Hospitals, Perungudi, Chennai, IND; 5 Orthopaedics, Adesh Medical College and Hospital, Ambala, IND; 6 Orthopaedics and Trauma, Osmania Medical College, Hyderabad, IND; 7 Orthopaedics and Trauma, Government Medical College, Bhadradri, Kothagudem, IND; 8 Orthopaedics, Pushpagiri Institute of Medical Sciences, Hyderabad, IND

**Keywords:** weight bearing, intertrochanteric hip fractures, hip fracture, dynamic hip screws, proximal femoral nails

## Abstract

Background

Intertrochanteric fractures, which occur in the hip of older individuals due to the weak and brittle structure of the bone caused by osteoporosis, make up over 50% of all hip fractures. There are several treatment options available for these fractures. The major objective of this study was to carry out a comparative analysis to evaluate the efficacy of dynamic hip screws (DHS) and proximal femoral nails (PFN) in treating intertrochanteric fractures.

Methodology

Two hundred instances of intertrochanteric hip fractures were surgically treated between July 2022 and January 2024 at a tertiary care facility. The evaluation of fractures was conducted in two groups, namely, group 1, which consisted of 140 patients, each having a fracture in one hip, treated using the DHS method. Group 2 consisted of 60 patients, each having a fracture in one hip, treated using the PFN technique. The evaluation of functional results was performed with the Harris hip score.

Results

In the investigation within these groups, group 1 produced excellent outcomes in 53 patients, which accounts for 37.86% of the total. In group 2, the expected results were achieved in 34 patients (56.67%). Achieved outcomes were favorable in 75 (53.57%) individuals in group 1 and 21 (35%) in group 2. Out of the individuals in group 1, eight (5.71%) saw benefits, whereas four (1.6%) did not gain significantly. In group 2, five (8.33%) individuals benefitted. None of the patients in group 2 had unfavorable outcomes.

Conclusion

While both PFN and DHS provide comparable outcomes in stable bone, PFN demonstrated superior results in cases of unstable bone. The use of PFN results in reduced surgical duration and a smaller surgical opening. Additionally, PFN exhibited superior specificity compared to DHS, especially in cases with stable intertrochanteric bone.

## Introduction

Intertrochanteric fractures make up over 50% of hip fractures in the elderly, with over 50% of these fractures being unstable [1]. The objective of treating intertrochanteric fractures is to safely and effectively restore mobility while reducing the likelihood of medical problems and returning the patient to their preoperative condition. The incidence of illness and death rises with advancing age [2]. The occurrence of trochanteric fractures is higher in females than in men, mostly owing to osteoporosis [2]. The literature has reported and used many surgical methods with various implants for treating intertrochanteric fractures. Historically, these types of fractures have received less attention due to their occurrence in porous bone with a very efficient blood supply, allowing them to mend naturally without requiring active intervention. However, conservative therapy led to the formation of a harmful thickened area of skin with inward angulation, outward rotation, and reduced length, resulting in an abnormal walking pattern and a significant risk of death owing to difficulties while laying down and protracted immobility. Some of the factors that increase the risk of these fractures include osteoporosis, poor diet, lack of exercise, impaired vision, neurological disorders, and imbalances in the muscles. In younger people, high-energy trauma is a common cause of intertrochanteric fractures. Various categories have been suggested, such as the Boyd and Griffin classification from 1949 [3], the Evans classification, and the AO classification [4,5].

The non-homogeneous structure and form of the bone, together with the complex stress arrangement, mostly impact the cortical and compact cancellous bone at this area when it breaks. The weakest link in the chain, the proximal femur, is where these breaks happen. Operative and non-operative methods are available for the treatment of intertrochanteric fractures [6]. In the early 19th century, the non-surgical approach was the preferred treatment strategy when the operating technology was not sufficiently advanced to provide secure fixation. Surgical intervention should be reserved for individuals who are unable to ambulate or suffer from persistent dementia and may effectively alleviate their discomfort with analgesics and rest. Additionally, this treatment is suitable for individuals who are diagnosed with terminal illnesses and have a life expectancy of less than six weeks, individuals with medical conditions that cannot be resolved through surgery, individuals with active infectious diseases that hinder the placement of surgical implants, and individuals with incomplete per trochanteric fractures as diagnosed by MRI [7].

Intertrochanteric fractures may be managed with either dynamic hip screws (DHS) or proximal femoral nails (PFN). The DHS is widely accepted and is regarded as the main choice and standard device for comparing results in stable or minimally displaced per trochanteric fractures. The DHS has shown efficacy in achieving positive outcomes; however, it is often accompanied by numerous problems, especially in cases of unstable intertrochanteric fractures. However, in cases of unstable fractures, the DHS device exhibits lower efficacy, with a considerably larger occurrence of failure in internal fixation [8].

PFN fixation has the benefit of creating a biomechanically stable structure by minimizing the gap between the hip joint and the implant. Additionally, it acts as a support to prevent sideways movement of the upper fragment. The placement of the connection between the nail and the lag screw inside the bone marrow makes the implant more resistant to bending forces [9,10]. An intramedullary device functions as a load-sharing device by bearing the bending force, which is then passed to the intramedullary nail. The nail resists this load by making contact with the medullary canal. This study aims to evaluate the clinical and radiological results of DHS and PFN in the treatment of intertrochanteric hip fractures, specifically focusing on the impact of weight-bearing on the outcomes.

## Materials and methods

Study design and setting

This was an observational comparative study conducted at a tertiary care hospital in north India. The study was carried out in the Department of Orthopedics in collaboration with the Department of Surgery and the Department of Anaesthesia for the surgical procedures. Consent was obtained or waived by all participants in this study. Institutional Ethics Committee for Research on Human Subjects of Adesh Medical College and Hospitals issued approval (RES/AMCH/ORTHO/2022).

Study participants

As per the hospital data for the instances of intertrochanteric fractures in the tertiary care center, a sample size of 180 was estimated. A total of 200 instances of intertrochanteric hip fractures were treated surgically between July 2022 and January 2024 at a tertiary care facility. The evaluation of fractures was conducted in two groups, namely, group 1, which consisted of 140 patients, each having a fracture in one hip, treated using the DHS method. Group 2 consisted of 60 patients, each having a fracture in one hip, treated using the PFN technique.

Patients with a follow-up period of less than one year for any orthopedic condition related to the hip joint, history of bilateral fractures (spontaneous), pathological fractures, fractures associated with various methods of injury, history of femoral malformation, and patients having subtrochanteric bone fractures or bone extensions up to 5 cm beyond the lower edge of the lesser trochanter, which hindered the use of hip nail osteosynthesis or intramedullary nail fixation, were not included in the study.

Data sources and measurements

For each of the 200 patients with intertrochanteric fractures, we noted gender, age at the time of fracture, fracture type (as per Arbeitsgemeinschaft für Osteosynthesefragen/Orthopedic Trauma Association (AO/OTA) classification), total working time (time from the beginning of the closure to the suturing of the wound), duration of treatment (confirmed by radiography), and the complications faced (early and late).

The choice of surgical procedure depended upon the surgeon's inclination and the specific implant being used. Prior to hip surgery, a uniform trauma team assessed all patients. The duration between the occurrence of the injury and the surgical procedure was 3.2 days, with a range of one to six days. The team implemented measures to guarantee that each individual gets optimal care during surgical procedures. The procedures were conducted using a traction table, and the closure reduction was verified using fluoroscopy in two distinct planes. The clinical outcomes of each group were examined, including functional impairment and both early and late complications. The patients were looked closely at in the first month. Patients were observed every four weeks, eight weeks, 12 weeks, six months, and then annually after that as a follow-up schedule. The Harris hip score was used to evaluate the functional results. Harris hip score rating was given a grading as per the points. Less than 70 points were graded as poor, 70-79 points as fair, 80-89 points as good, and 90-100 points as excellent.

Data analysis

To identify any differences between the two surgical techniques, they were compared across all research variables. IBM SPSS Statistics 25.0 (IBM Corp., Armonk, NY) was used to examine the data. Means, standard deviations, medians, and percentages were used to assess and define the study variables. Independent sample t-tests were used to compare the outcome variables between the two surgical procedures. For statistical purposes, a p-value less than 0.05 was considered significant.

## Results

The average age of the patients included in our research was 58.34 ± 6.38 years, with a range of 18 to 77 years. There was no statistically significant difference in age between group 2 and group 1 (p = 0.18). Our research showed a predominance of females, with 60% of the overall cases being female. The predominant cause of injury was a minor fall, specifically a fall with little damage, which included 115 (57.5%) of all reported cases. Out of the total fractures, 137 (68.5%) were stable fractures and 63 (31.5%) were unstable fractures. The preoperative comparisons are succinctly shown in Table 1.

**Table 1:** Basic parameters of the participants The details of basic parameters, along with the percentage and the p-value, have been depicted in the two groups. A p-value less than 0.05 was considered significant.

Parameter	Group 1	Group 2	Total		P-value
	Number = 140	Number = 60	Number	Percentage	
Gender					0.12
Female	80	40	120	60%	
Male	60	20	80	40%	
Age					0.18
Below 30	3	2	5	2.5%	
30-40	22	9	31	15.5%	
40-50	34	18	52	26%	
50-60	73	29	102	51%	
Above 60	8	2	10	5%	
Mean age ± standard deviation	59.35 ± 11.87	57.16 ± 7.76			
Fracture pattern					0.16
A1	100	10	110	55%	
A2	25	20	45	22.5%	
A3	15	30	45	22.5%	
Fracture type					0.21
Stable	97	40	137	68.5%	
Unstable	43	20	63	31.5%	
Mode of injury					0.15
Trivial fall	80	35	115	57.5%	
Road traffic accident	32	12	44	22%	
Fall from height	28	13	41	21.5%	

The mean surgical length was longer in group 1 (64.09 ± 7.78 minutes) compared to group 2 (60.34 ± 6.45 minutes), as shown in Table 2, but this difference was not statistically significant. In this study, group 1 had a much more significant mean blood loss than group 2, with four patients in group 1 needing blood transfusion postoperatively, while none in group 2 did. The difference in blood loss between the two groups was statistically significant (p < 0.001). There were three instances requiring open reduction in both group 2 and group 1, while all other cases were significantly reduced. The length of hospitalization was similar. Radiological union occurred at an average size of 14.99 ± 2.56 weeks for group 1 and 14.04 ± 2.37 weeks for group 2, which were identical.

**Table 2:** Perioperative and follow-up comparison The mean and standard deviation of preoperative and follow-up details in both groups have been depicted. The t-test was applied, and a p-value was derived, which was also added; a p-value less than 0.05 was considered significant.

	Group 1 (n = 140)		Group 2 (n = 60)		t-value	p-value
	Mean	Standard deviation	Mean	Standard deviation		
Duration of surgery (minutes)	64.09	7.78	60.34	6.45	1.23	0.21
Blood loss (ml)	277.89	12.88	205.89	11.56	4.22	<0.001
Hospital stay (days)	7.65	1.06	7.33	1.44	0.34	0.27
Radiological union (weeks)	14.99	2.56	14.04	2.37	0.78	0.19
Harris hip score	82.93	5.77	85.34	5.39	0.89	0.11

Patients in group 1 had better outcomes than those in group 2 when examining a subset comparing radiological union and Harris hip score of unstable fractures, and the same has been depicted in Table 3.

**Table 3:** Subgroup analysis

Group 1	Radiological union		Harris hip score	
Stable	15.76	2.14	85.22	5.37
Unstable	17.21	2.65	77.11	4.38
Group 2				
Stable	14.23	2.11	86.16	5.28
Unstable	15.11	3.98	82.45	5.12

In the investigation within these groups, group 1 produced excellent outcomes in 53 patients, which accounts for 37.86% of the total. In group 2, outstanding results were achieved in 34 patients (56.67%). Achieved outcomes were favorable in 75 (53.57%) individuals in group 1 and 21 (35%) in group 2. Out of the individuals in group 1, eight (5.71%) saw benefits, whereas four (1.6%) did not gain significantly. In group 2, five (8.33%) individuals benefitted. None of the patients in group 2 had unfavorable outcomes. These outcomes have been tabulated in Table 4.

**Table 4:** Functional outcomes Functional outcomes in terms of number and percentage have been depicted.

	Group 1		Group 2	
	Number	Percentage	Number	Percentage
Excellent	53	37.86%	34	56.67%
Good	75	53.57%	21	35%
Fair	8	5.71%	5	8.33%
Poor	4	2.86%	0	0%

The early consequences seen were persistent discharge, blood clot formation, infection in the face, and the development of a blood clot in a deep vein. Notable late consequences include growth impairment, irregularity, graft rejection, and delayed infection, as shown in Table 5.

**Table 5:** Complications The complications faced by the patients and the number of patients facing them have been described.

	Group 1 (n = 140)	Group 2 (n = 60)
Early	Number of participants facing the complication	Number of participants facing the complication
Chronic drainage	3	0
Hematoma	3	0
Facial infection	5	3
Deep vein thrombosis	2	0
Late		
Stunting	4	0
Varus malunion	4	2
Inconsistency	3	1
Transplant failure	3	0
Infection	4	0

## Discussion

The most important finding(s) of the study was the varied response rates among the two groups examined. Specifically, group 1 saw excellent outcomes in 53 patients, accounting for 37.86% of the group, with favorable results in 75 patients (53.57%). In contrast, group 2 achieved expected results in 34 patients, or 56.67% of its total, with 21 individuals (35%) experiencing favorable outcomes. Additionally, in group 1, eight individuals (5.71%) saw benefits, whereas in group 2, only four (1.6%) saw minimal gains.

Fractures in the intertrochanteric area of the femur provide a significant challenge for orthopedic specialists. The goal is not only to achieve proper healing of the fractures but also to restore maximal function as quickly as possible while minimizing consequences. The primary objective of fracture care has shifted toward achieving prompt mobility, accelerated rehabilitation, and swift reintegration of persons into their pre-injury home and work environments, functioning independently both physically and mentally. Operative or surgical therapy, namely, internal fixation, allows for early rehabilitation and provides the highest likelihood of functional recovery. As a result, it has become the preferred and most effective treatment for almost all fractures in the intertrochanteric area. The compression hip screw is the most popular and well-regarded implant among several options, including intramedullary devices, fixed nail plate devices, and sliding nail or screw plates. However, closed intramedullary nailing techniques have recently become very popular. The management of intertrochanteric hip fractures remains a significant orthopedic difficulty, with ongoing discussions in the literature on the choice of implant for such fractures. Several randomized controlled trials have shown statistically significant benefits of PFN compared to DHS for treating unstable fractures in elderly patients with co-morbidities [11,12]. The PFN was developed in the early 1990s to improve the surgical treatment of unstable intertrochanteric fractures. It offers biomechanical and biological advantages over the DHS. The PFN is an intramedullary device that mitigates the effects of a medialization of the shaft by compensating for the medial column's function, reducing the bending force, and keeping the fracture site from becoming much shorter [13,14]. The average age of the patients in our research was 58.34 ± 6.38 years. The trochanteric area is the most prevalent location for senile osteoporosis as individuals age. Due to its role in weight-bearing, the hip joint, being a significant joint, is unable to tolerate unexpected aberrant stress, especially when it is already compromised. The intertrabecular gap is expanded and filled with adipose tissue, while the surrounding compact tissue is reduced in thickness, and the calcar is undergoing atrophy. Because intertrochanteric fractures are fixed early and patients are mobilized early, they may achieve a complete range of motion soon after the injury with no impact on their production.

Our analysis closely aligns with the study conducted by Sharma et al., which reported an average age of 61.47 years [15]. Group 2 had a shorter duration of surgery by an average of 3.75 minutes; however, this difference was not statistically significant. Similar results were found by Saudan et al., who saw a mean difference of one minute. The results of Baumgaertner et al. were inconsistent with this investigation since they reported considerably longer surgery times in group 1 in their series [8,16]. The average length of hospitalization was shorter in group 2. The patients in group 1 saw a notably greater amount of blood loss during surgery, namely, 72 milliliters or more, compared to group 2. Baumgaertner et al., Zhao et al., and Zhang et al. similarly observed a markedly greater amount of blood loss during surgery in group 1 [16-18].

In contrast to the results of this study, Pajarinen and colleagues discovered no statistically significant disparity in blood loss between the two groups [19]. In both cases, fractures were fully healed at an average of 15.76 ± 2.14 weeks for DHS and 14.23 ± 2.11 weeks for PFN. When comparing the union time for unstable fractures individually, it was shown that group 2 had a superior fracture union time (15.11 ± 3.98 weeks) compared to group 1. This study was similar to the studies conducted by Herode et al. and Klinger et al. [20,21]. The functional result of patients treated with PFN was marginally superior to that of patients treated with DHS; however, this difference was not statistically significant (p = 0.13). However, upon an independent comparison of stable and unstable fractures, we discovered that the functional result of unstable fractures treated with group 2 was significantly superior to that of group 1. The average Harris hip score for group 2 was 82.93 ± 5.77, whereas group 1 had an average score of 85.34 ± 5.39. In the investigation of these options, group 1 got excellent outcomes in 53 patients, accounting for 37.86% of the total. In contrast, group 2 had excellent results in 34 patients, representing 56.67% of the total. Achieved outcomes were favorable in 75 (53.57%) individuals in group 1 and in 21 (35%) individuals in group 2. Out of the individuals in group 1, eight (5.71%) saw benefits, whereas four (1.6%) did not gain significantly. In group 2, five (8.33%) individuals benefitted. Group 2 did not have any patients with unfavorable findings. In a comparison research conducted by Gill et al., including 80 patients who used the locking DHS and PFN, it was shown that in the DHS group, six patients (15%) achieved great results, 14 patients (35.0%) achieved good results, 12 patients (30.0%) achieved acceptable results, and eight patients (20.0%) had bad results. The PFN group demonstrated outstanding outcomes in 20.0% of cases, while 75.0% of cases showed good results. Only 5.0% of cases had fair results, and no bad results were seen [22].

In their research, Parikh et al. [23] observed that out of the 52 patients in the DHS group, six (23%) had outstanding outcomes, five (19%) achieved good results, 13 (50%) achieved medium results, and two (8%) achieved bad results. The PFN group exhibited outstanding outcomes in four cases (15%), satisfactory outcomes in 14 cases (54%), moderate outcomes in seven cases (27%), and unsatisfactory outcomes in one case (4%).

In their research of 30 patients, Harish et al. [24] observed that in the DHS group, six patients (50%) had great outcomes, two patients (13.33%) achieved good results, two patients (13.33%) achieved fair results, and no patients had bad results. Within the PFN group, a majority of eight cases (72.73%) showed great outcomes, one case (9.1%) showed good results, one case (9.1%) showed fair results, and no cases exhibited bad results.

The findings were similar to the research conducted by Giraud et al. and Karanam and colleagues [25,26]. Contrary to our research, Mavrogenis et al. and Mereddy et al. found that PFN had worse functional outcomes compared to DHS [27,28]. Our research indicates that PFN may be the optimal choice for treating unstable fractures when compared to DHS. However, more study is necessary to carry out a thorough statistical analysis of subgroups. Out of the patients in the DHS group, three people had a superficial wound infection, while one patient had a protracted duration of drainage. By contrast, the PFN group had a superficial wound infection in just two individuals. However, all people responded well to local debridement and antibiotics based on culture and sensitivity tests. The extraction of the implant was deemed unnecessary due to the absence of any infection. There was no statistically significant disparity in infection rates seen between the two groups. This conclusion is consistent with the results of Herode et al. and Saudan et al. [8,20].

The research was limited by a lower sample size and a shorter follow-up time. The outcome measures are straightforward and there is stratification present. Despite these limitations, the outcomes of this study remain optimistic. Nevertheless, there were minor disparities observed across some variables, which might potentially become statistically significant when analyzed with a sufficiently large sample size. Therefore, more research is required over an extended duration, using a sufficiently big sample size, to conduct a subgroup analysis.

## Conclusions

While both PFN and DHS provide comparable outcomes in stable bone, PFN demonstrates superior results in cases of unstable bone (intertrochanteric fractures), and using PFN results in reduced surgical duration and a smaller surgical opening. Additionally, PFN exhibits superior specificity compared to DHS, especially in cases with stable intertrochanteric bone.
